# Probiotic supplementation and risk of necrotizing enterocolitis and mortality among extremely preterm infants—the Probiotics in Extreme Prematurity in Scandinavia (PEPS) trial: study protocol for a multicenter, double-blinded, placebo-controlled, and registry-based randomized controlled trial

**DOI:** 10.1186/s13063-024-08088-8

**Published:** 2024-04-12

**Authors:** Sofia Söderquist Kruth, Carl Willers, Emma Persad, Elisabeth Stoltz Sjöström, Susanne Rautiainen Lagerström, Alexander Rakow

**Affiliations:** 1 Women’s Health and Allied Health Professional Theme, Karolinska University Hospital, Solna, 17176 Stockholm, Sweden; 2 Department of Women’s and Children’s Health, Karolinska Institutet, 17177 Stockholm, Sweden; 3 Department of Neurobiology, Care Sciences and Society, Karolinska Institutet, 14152 Huddinge, Sweden; 4 Department of Food, Nutrition and Culinary Science, Umeå University, Umeå, Sweden; 5 Department of Global Public Health, Karolinska Institutet, Stockholm, Sweden; 6 K2 Medicin, Solna, Karolinska Institutet, Stockholm, Sweden; 7 Department of Neonatology, Karolinska University Hospital, Solna, 17176 Stockholm, Sweden

**Keywords:** Probiotics, Necrotizing enterocolitis, Extreme prematurity, Mortality, Feeding tolerance, Growth failure, Randomized controlled trial

## Abstract

**Background:**

Extremely preterm infants, defined as those born before 28 weeks’ gestational age, are a very vulnerable patient group at high risk for adverse outcomes, such as necrotizing enterocolitis and death. Necrotizing enterocolitis is an inflammatory gastrointestinal disease with high incidence in this cohort and has severe implications on morbidity and mortality. Previous randomized controlled trials have shown reduced incidence of necrotizing enterocolitis among older preterm infants following probiotic supplementation. However, these trials were underpowered for extremely preterm infants, rendering evidence for probiotic supplementation in this population insufficient to date.

**Methods:**

The Probiotics in Extreme Prematurity in Scandinavia (PEPS) trial is a multicenter, double-blinded, placebo-controlled and registry-based randomized controlled trial conducted among extremely preterm infants (*n* = 1620) born at six tertiary neonatal units in Sweden and four units in Denmark. Enrolled infants will be allocated to receive either probiotic supplementation with ProPrems® (*Bifidobacterium infantis*, *Bifidobacterium lactis*, and *Streptococcus thermophilus*) diluted in 3 mL breastmilk or placebo (0.5 g maltodextrin powder) diluted in 3 mL breastmilk per day until gestational week 34. The primary composite outcome is incidence of necrotizing enterocolitis and/or mortality. Secondary outcomes include incidence of late-onset sepsis, length of hospitalization, use of antibiotics, feeding tolerance, growth, and body composition at age of full-term and 3 months corrected age after hospital discharge.

**Discussion:**

Current recommendations for probiotic supplementation in Sweden and Denmark do not include extremely preterm infants due to lack of evidence in this population. However, this young subgroup is notably the most at risk for experiencing adverse outcomes. This trial aims to investigate the effects of probiotic supplementation on necrotizing enterocolitis, death, and other relevant outcomes to provide sufficiently powered, high-quality evidence to inform probiotic supplementation guidelines in this population. The results could have implications for clinical practice both in Sweden and Denmark and worldwide.

**Trial registration:**

(Clinicaltrials.gov): NCT05604846

**Supplementary Information:**

The online version contains supplementary material available at 10.1186/s13063-024-08088-8.

## Introduction

### Background and rationale {6a}

Despite considerable improvements in preterm infant care in the last decade, approximately 20% of extremely preterm infants, defined as those born before 28 weeks’ gestational age, do not survive past the neonatal period in Sweden [1]. Extremely preterm infants are at a considerably high risk for serious adverse outcomes, including necrotizing enterocolitis (NEC), late-onset sepsis (LOS), and death [2]. Necrotizing enterocolitis, an inflammatory gastrointestinal disease, is regarded as the most severe gastrointestinal-related morbidity in preterm infants and has a high mortality rate [3, 4]. Due to the severity and high incidence of the disease in preterm infants, recent research has focused on preventative measures to combat the development of NEC. One such measure is the supplementation of probiotics, which is thought to improve gastrointestinal tolerance and colonization of beneficial bacteria in these infants, ultimately helping their microbiota resemble that of a healthy infant. To date, preemptive provision of probiotics for NEC prevention has gained immense clinical and research interest worldwide.

The Probiotics in Extreme Prematurity in Scandinavia trial (PEPS trial) is comprised of two parts. The first part is a multicenter trial which aims to investigate the effect of probiotic vs. placebo supplementation on the incidence of NEC and death. This cohort will hereafter be referred to as the “complete cohort.” The second part aims to assess the effect of probiotic supplementation on feeding tolerance, postnatal growth, and body composition. This analysis will solely be conducted on a cohort of infants at Karolinska University Hospital in Stockholm, Sweden, due to limited availability of feeding and growth data at other sites. This population will hereafter be referred to as the “sub-cohort.”

A significant number of randomized controlled trials (RCTs), systematic reviews, and meta-analyses have been conducted on the effect of probiotic supplementation in preterm infants [5–27]. However, no RCT has specifically investigated supplementation in extremely preterm infants, and review articles published to date generally analyze infants born before gestational week 32. Consequently, any analyses on infants born before gestational week 28 have generally been subgroup analyses older cohorts, rendering the results potentially imprecise for the extremely preterm population. Furthermore, wide variation in probiotic strains, dosages, and duration of supplementation complicate comprehensive analyses [28]. As such, a sufficiently large trial focused on extremely preterm infants using only one probiotic product is warranted.

## Risk of morbidity and mortality (complete cohort)

### Pathogenesis of NEC

Despite considerable investigative research to date, the pathogenesis of NEC is not well understood. The nature of the disease is widely accepted to be multifactorial, particularly owing to the general immaturity of the gastrointestinal system, namely the intestinal mucosa barrier function. This lack of robustness from the immature gastrointestinal system, alongside the disarray caused by the infiltration of pathogenic bacterial species and exposure to harmful ischemic events, is proposed to instigate the development of NEC [3, 4, 29]. The considerably higher rates of NEC in premature infants compared to those born at term are further evidence for the role of immaturity and disarray in this disease. Several studies have also postulated that the routine use of prophylactic antibiotics in this population negatively impacts the diversity of the microbiome, rendering infants more vulnerable to the overgrowth of harmful bacteria species and eventual NEC development [30–32]. This highlights the immense potential for prevention through strengthening the diversity of the microbiome via probiotic administration.

The current conservative treatment for NEC is bowel rest through parental nutrition and administration of antibiotics for approximately 10 to 14 days. Gastrointestinal surgery is required in up to 50% of cases [3]. Infants surviving NEC may be subject to long-term adverse effects and complications, such as impaired growth, intestinal strictures, or even short-gut syndrome [33, 34]. These drastic treatment measures and considerable risk of morbidity highlight the necessity of further research into preventing NEC.

### Pathogenesis of late-onset sepsis

Extremely preterm infants are also at high risk for late-onset sepsis (LOS), defined as sepsis occurring 72 h after birth. Well known risk factors include an immature skin-mucosal barrier, immature immune response, prolonged duration of parenteral nutrition, prolonged hospitalization, previous surgery, and underlying respiratory and cardiovascular diseases [30, 31].

### Antibiotic treatment

Once NEC or LOS is suspected or confirmed, immediate antibiotic treatment is routinely started. According to the Swedish Neonatal Quality Register (SNQ), the mean duration of antibiotic treatment for extremely preterm infants is around 25 days [1]. Previous research has found that infants receiving antibiotic treatment during the first week of life have an altered gut microbiome with lower bacterial diversity, the extent of which was recently explored in a study of 32 preterm infants [32, 35]. Consequently, antibiotic-induced microbiome alterations have been linked to gastrointestinal diseases in preterm infants and developmental growth changes in term infants, including higher body mass indices (BMI) [36, 37]. Hence, antibiotics used for the treatment of NEC and LOS may also be associated with short- and long-term health consequences in preterm infants.

### Probiotics as a nutritional intervention strategy in preterm infants

Despite lack of consensus on methods to prevent NEC, the gut microbiome may work as a preventative target due to the potential to influence the immature gastrointestinal system in extremely preterm infants. In these infants, intestinal bacterial colonization with normal and beneficial bacterial flora (*Bifidobacteria*) is often delayed, rendering the microbiota less diverse and dominated by *Enterobacteriacea*, increasing the potential for the invasion of harmful species [38].

Probiotics are living microorganisms and adequate administration has been linked to numerous benefits, including increased stimulation of immune functions, inhibition of pathogenic bacteria, degradation and fermentation of certain foods, and production of fat-soluble vitamins [39, 40]. The effectiveness of probiotic supplementation in modifying the gut microbiome and preventing NEC and LOS has been shown in recent research; however, the evidence among extremely preterm infants is limited. Further, evidence backing the routine use of probiotics has been challenged due methodological constraints, namely inconsistencies regarding type of probiotics tested, dosage given, enteral feeding routine, intervention duration, outcome measure definition, and general heterogeneity between studies, rendering it difficult to draw sound conclusions on effectiveness [28, 41, 42]. Nevertheless, the recent position paper, “*Probiotics and Preterm Infants*” by the European Society for Paediatric Gastroenterology, Hepatology and Nutrition (ESPGHAN), conditionally recommended probiotic supplementation in infants with birth weight <1500 g if certain safety measures are achieved [15]. The committee recommended providing either *L. rhamnosus GG ATCC53103* or a combination preparation of *Bifidobaterium infantis Bb-02*, *Bifidobacterium lactis Bb-12*, and *Streptococcus Thermophilus TH4*, the latter of which will be used in our study.

### The preventative effect of B. infantis Bb-02, B. lactis Bb-12, and Str. Thermophilus TH4 on NEC and mortality

Two RCTs have investigated the combination preparation of *B. infantis Bb-02*, *B. lactis Bb-12*, and *Str. Thermophilus TH4* in infants <1500 g [12, 13]. One study enrolling 145 infants reported a reduction in both incidence and severity of NEC and death as a composite outcome [13]. They also found that all-cause mortality was significantly lower in the intervention group, but no difference in the incidence NEC-specific death. Following this, *The ProPrems Trial* conducted in Australia and New Zealand in 2012 investigating 1099 very preterm infants reported a significant reduction in incidence of NEC; however, no significant effect on mortality or LOS [12]. In a systematic review and meta-analysis from 2021 summarizing 45 trials including 12,320 infants, a combination of *Bifidobacterium* and *Lactobacillus* was found to be associated with a 54% lower rate of NEC and a 44% lower rate of all-cause mortality [43]. To date, there is only one RCT assessing the preventative effect of *B. infantis Bb-02*, *B. lactis Bb-12*, and *Str. Thermophilus TH4* via subgroup analysis, which found only a weak, non-significant association between probiotic supplementation and reduced risk of NEC [12]. Another RCT included infants between 500 and 1000 g and found no statistically significant difference in NEC reduction between groups following the administration of their single strain probiotic [44].

In a resent observational study, the incidence of NEC was lower in the group receiving probiotics (adjusted odds ratio (OR) 0.64, 95% confidence interval (CI) 0.41; 0.996) [45]. However, only one of the three strains of interest to our study was used. Subgroup analysis showed a significant reduction in NEC (adjusted OR 0.52, 95% CI 0.31; 0.87) or NEC and mortality (adjusted OR 0.34, 95% CI 0.22; 0.52). Another observational study reported that the adjusted risk estimate for NEC was not beneficially affected by probiotic supplementation. However, a subgroup analysis found a statistically significant reduction in NEC for the group receiving probiotics [46]. The study used one of the strains that will be used in the PEPS trial.

### The preventative effect of B. infantis Bb-02, B. lactis Bb-12, and Str. Thermophilus TH4 on LOS

No effect on LOS incidence was observed using the combination preparation of *B. infantis Bb-02*, *B. lactis Bb-12*, and *Str. Thermophilus TH4* in two RCTs [12, 13]. Comparatively, a strain-specific systematic review and meta-analysis including 51 RCTs with data on 11,231 infants and 25 different probiotic treatments identified two studies reporting on a significant beneficial effect on LOS incidence [19]. Similarly, a systematic review and meta-analysis including 25 RCTs found a significant reduction in LOS in human-milk-fed preterm infants [21]. RCTs investigating the effect of probiotics on neonatal LOS solely in extremely preterm infants are lacking.

### Study rationale

Studies have shown conclusive results on the effect of the combination of *B. infantis Bb-02*, *B. lactis Bb-12*, and *Str. Thermophilus TH4* in reducing NEC incidence in preterm infants born after 28 gestational weeks [12, 13]. However, previous research has been underpowered for extremely preterm infants, rendering the efficacy in this group not fully understood or supported. Consequently, the Swedish national guidelines outlining neonatal probiotic supplementation only recommend supplementation to infants older than 28 weeks of gestation, not those born extremely preterm. Unfortunately, this subgroup of infants has the highest incidence of mortality, NEC, and other neonatal morbidities, highlighting the need for evidence on the effectiveness of preventive strategies to reduce morbidity and mortality.

The PEPS trial aims to fill this knowledge gap, providing high-quality evidence on probiotic supplementation in extremely preterm infants, potentially informing Swedish national guidelines on routine probiotic administration in this group of infants. The results will have implications for this population both in similar settings and also globally.

## Feeding tolerance and postnatal growth (sub-cohort)

The following outcomes will be investigated in a sub-cohort of infants from Karolinska University Hospital in Stockholm, Sweden.

### Postnatal growth failure

Extrauterine growth restriction (EUGR) is a state of postnatal growth failure during the first postnatal weeks. Despite intensive nutritional management, it is common among extremely preterm infants [47]. Receiving the recommended dietary nutrients is essential for minimizing the risk of EUGR [48, 49], particularly as postnatal growth failure influences long-term outcomes [50–54]. In particular, head circumference growth strongly correlates to brain volume, which is important for neurological development [50–52], while weight gain and increase in length affect lung size and maturity [53, 54]. Thus, healthy growth is crucial for the health and development of extremely preterm infants. Comparatively, extreme weight gain is associated with a higher risk of obesity and cardiovascular diseases later in life due to a higher ratio of fat mass, highlighting the necessity for a balanced intake of required nutrients [55, 56]. Interestingly, a higher ratio of fat-free mass in one study was associated with improved neurological development [57]. Preterm infants have previously been shown to be at high risk of developing a disadvantageous distribution of fat; however, few extremely preterm infants were included in these studies, rendering further investigation [58, 59].

### Feeding tolerance

Feeding tolerance is essential to maintain adequate nutritional intake and promote growth in preterm infants. However, a common complication for extremely preterm infants is intolerance to enteral nutrition, leading to prolonged withholding of feedings and delays to full enteral feeds [60–63]. Signs of feeding intolerance include gastric residuals, vomiting, and distended abdomen and are often associated with apnea and bradycardia. The combination of these symptoms usually raises clinical suspicion for NEC or LOS, generally leading to fasting. There is also an uncertainty about when to restart feeding after fasting periods and at what rate and volume [64]. Slow acceleration of enteral feeds and small volumes are often used as precautionary methods, which may prolong the time of inadequate enteral nutrition, facilitating EUGR [61]. Preventive measures and medications to improved feeding tolerance are therefore important in the nutritional management of extremely preterm infants.

### Probiotics and feeding tolerance

As NEC and LOS are often the main outcome measures in probiotic studies, the effect of probiotics on feeding tolerance and developmental outcomes are not well understood [6, 9, 65, 66]. Further, in studies exploring these outcomes, the infants are often of higher gestational age. A systematic review and meta-analysis including 1244 infants concluded that probiotics may promote growth and improve feeding tolerance in extremely preterm infants [66]. However, the heterogeneity in supplementation, dosage, and wide gestational age range rendered conclusions difficult to draw.

A pre-post implementation study conducted in Sweden considered data before and after the combination of *B. infantis Bb-02*, *B. lactis Bb-12*, and *Str. Thermophilus TH4* had been introduced into clinical routine for infants born after 28 gestational weeks [67]. In this study, fewer days until full enteral feeds, improved growth, and reduced risk of NEC and LOS were observed. However, these results should be interpreted with caution due to the retrospective pre-post implementation study design.

As nutritional status and postnatal growth is vital for the care and development of extremely preterm infants, we aim to investigate the role of probiotic supplementation in relation to feeding tolerance, nutritional intake, postnatal growth, and body composition using a sub-cohort of the study population.

### Objectives {7}

The overall aim is to determine whether early probiotic supplementation can be used as a preventative nutritional intervention to reduce the risk of NEC and neonatal mortality among extremely preterm infants born before 28 weeks of gestational age. The effect of supplementation on other common neonatal morbidities will also be studied.

#### Primary aim

1. To determine whether probiotic supplementation will reduce the incidence of composite endpoint NEC and/or neonatal mortality in extremely preterm infants.

#### Secondary aims

1. To determine whether probiotic supplementation will reduce the incidence of NEC.
2. To determine whether probiotic supplementation will reduce the incidence of neonatal mortality.
3. To determine whether probiotic supplementation will reduce the incidence of late-onset sepsis.
4. To determine whether probiotic supplementation will reduce the rate and duration of antibiotic use during hospital stay.
5. To determine whether probiotic supplementation will reduce duration of hospital stay.
6. To determine whether probiotic supplementation has an effect on the development and composition of the microbiome.

#### Tertiary aims

1. To assess whether probiotic supplementation reduces the incidence of enteral feeding intolerance in a sub-cohort of extremely preterm infants.
2. To assess whether probiotic supplementation as an influence on postnatal growth and body composition after discharge in a sub-cohort of extremely preterm infants.

### Trial design {8}

The PEPS trial is a multicenter, double-blinded, placebo-controlled, and registry-based RCT with two parallel arms. There are two parts of the study: one evaluating risk of NEC and/or mortality in the whole cohort, and the other evaluating feeding tolerance and postnatal growth in a sub-cohort at Karolinska University Hospital. The PEPS trial is a superiority study with an even distribution to (1:1) probiotic or placebo supplementation. The PEPS trial is designed in accordance with recommendations for interventional trials [68] and Consolidated Standards for Reporting of Trials CONSORT guidelines [69].

## Methods: participants, interventions, and outcomes

### Study setting {9}

We will include 1620 extremely preterm infants at gestational age 22 weeks + 0 days to 27 weeks + 6 days. The infants will be recruited between approximately December 2022 and July 2026 or until enough participants have been included.

Infants will be recruited at six university hospitals in Sweden (Karolinska University Hospital, Skåne University Hospital, Sahlgrenska University Hospital, Linköping University Hospital, Uppsala Univesity Hospital, and Umeå University Hospital), and four university hospitals in Denmark (Rigshospitalet Copenhagen/Glostrup, Aarhus University Hospital, Aalborg University Hospital, and Odense University Hospital).

A sub-study conducted on the sub-cohort will be performed to assess feeding tolerance and postnatal growth. Infants recruited at Karolinska University Hospital will be included in this extended analysis.

### Eligibility criteria {10}

#### Inclusion criteria

- Extremely preterm infants (born before 28 completed gestational weeks)
- Infants enrolled within 72 h after birth with informed consent
- Infants born at Karolinska University Hospital (sub-cohort)

#### Exclusion criteria

- Infants with severe complications with low chance of survival detected within 72 h after birth
- Infants with major congenital anomalies
- Infants participating in another interventional trial where main outcome includes incidence of NEC

### Informed consent {26a}

Legal guardians of eligible infants meeting the aforementioned criteria will be approached by clinicians and/or a study nurse during the first 48 h postpartum. The legal guardians will be provided verbal and written information prior to participation. Information can also be given antenatally when suitable. Informed signed consent from at least one legal guardian must be obtained within 72 h after birth. Signed consent can be retrieved from both clinicians and study nurses but must later be signed by the principal investigator (PI) at each hospital.

Legal guardians can choose to withdraw participation without giving a reason at any time. If the legal guardians choose to interrupt the infant’s study participation, this will not affect the infant’s continued care and treatment.

### Additional consent provisions for collection and use of participant data and biological specimens {26b}

The effect of probiotics on the gut microbiome will be investigated through stool sample analysis. The informed consent form explains the process of stool sample collection, biobank storage, and future analysis plans. If the legal guardians do not consent to the collection of samples and/or saving these in a biobank, the participating infant will still be eligible to be included in the study without providing stool samples.

## Interventions

### Choice of probiotic {6b}

The PEPS trial will us the probiotic supplement known commercially as ProPrems®, which is comprised of a combination of one billion freeze-dried bacteria per 0.5 g in a maltodextrin base powder (*Bifidobacterium infantis Bb-02* (DSM 33361) 300 million, *Bifidobacterium lactis* (BB-12®) 350 million, and *Streptococcus thermophilus* (TH-4®) 350 million). The probiotic supplement is one of two probiotic combinations recommended by the ESPGHAN committee [15]. The product is produced by Chr Hansen in Denmark and distributed by the Swedish company Neobiomics©. The product is currently being used routinely in all Swedish hospitals for infants born in gestational weeks 28 to 32. Thus, the PEPS trial will be easy to implement considering the product is already familiar to medical and nursing staff at the participating hospitals. However, participating Danish hospitals have not implemented any probiotic supplement into clinical routine and will need to learn these routines. The control group will receive placebo produced by Chr Hansen containing 0.5 g maltodextrin powder, equivalent to the amount of maltodextrin included in the probiotic supplement.

### Intervention description {11a}

The probiotics (intervention) or placebo (control) will be mixed with breastmilk and administered by gastric tube when infants tolerate at least 3 mL breastmilk per meal. The breastmilk may however be administered as a bolus or continuous feeds. For the intervention group, the standard dose of 0.5 g will be mixed with 3 mL breastmilk. For the control group, 0.5 g of placebo containing only maltodextrin powder will be used. If the mother’s breastmilk is available, this will be primarily used; otherwise, donated breastmilk will be given. There will be no visible difference between the breastmilk containing probiotic supplementation and placebo. The dose will be administered as the first meal of the day once daily until the infant reaches 34 weeks of gestation. The dosage and method of administration will not change with increasing age and weight. If the infant does not tolerate the enteral feeds after the start of probiotic supplementation and the administrated volume drops below 3 mL, the supplementation will be paused and started again when the infant tolerates 3 mL per meal again. If the infant does not receive the planned dose, this must be noted in the case report form (CRF). Unopened probiotic supplements and placebo will be stored at room temperature in a nutrition kitchen.

### Criteria for discontinuing or modifying the allocated interventions {11b}

The supplementation of probiotics (intervention) or placebo (control) will be administered in the same manner as given in the ProPrems trial [12] and according to existing clinical routines previously mentioned for infants born after gestational week 28 in Swedish hospitals. Variation in the timing of the administration of the intervention is acceptable if local clinical routines for probiotic supplementation are already set. As the probiotic mixture has been reported to remain stable for 4 h, the intervention must be given within 4 h [70]. Otherwise, a new mixture must be prepared.

All researchers, medical, and nursing staff will be blinded until the final analysis has been performed. However, code breaking will occur in connection to a suspected unexpected serious adverse reaction (SUSAR) due to the seriousness of the situation.

### Strategies to improve adherence to interventions {11c}

All infants will be recruited as inpatients and the intervention will be completed before discharge. Thus, the compliance to intervention does not rely on legal guardians’ adherence to instructions, rather to research and health care personnel at the clinic.

### Relevant concomitant care permitted or prohibited during the trial {11d}

Infants participating in another intervention trial where main outcome includes incidence of NEC cannot be included in this trial, as stipulated in the exclusion criteria. Patient insurance applies to infants in the study, as with all other care during hospital admission, and all study participants will be treated according to the existing standard of care beyond the studied intervention.

### Provisions for post-trial care {30}

There is no anticipated need for post-trial care. Patients will receive adequate care during and after hospital admission irrespective of their allocation to the intervention or control group. All extremely preterm infants are followed up at a neonatal ward until 5 years of age in both Sweden and Denmark as per clinical routine.

### Outcomes {12}

Table 1 (complete cohort) and 2 (sub-cohort) include descriptions of primary, secondary, and other outcomes including analysis metric, method of aggregation and time points for outcome collection in the PEPS trial. Details of NEC and LOS will be collected for validation of diagnoses and understanding of disease events. Serious adverse events (SAE) will be collected for interim analysis. All covariates collected in the trial are relevant for analyzing the risk of developing the primary and secondary outcomes and will primarily be used for sensitivity analysis.

**Table 1:** Description of outcomes with analysis metric, aggregation method, and time points in main cohort

Outcome variables complete cohort			
*Primary outcome variables*	*Analysis metrics*	*Method of aggregation*	*Time point*
Necrotizing enterocolitis	Incidence rate	Mean/Median	During study intervention (until 34 weeks of gestational age)
Necrotizing enterocolitis Bell Stage >II^a^ Verified	Incidence rate	Mean/Median	During study intervention (until 34 weeks of gestational age)
Necrotizing enterocolitis Bell Stage >III^a^ Verified	Incidence rate	Mean/Median	During study intervention (until 34 weeks of gestational age)
Necrotizing enterocolitis Operation	Date, time	Mean/Median	During study intervention (until 34 weeks of gestational age)
Abdominal symptoms	Incidence of free gas, intermural intestinal gas, inflated abdomen discolored abdomen, stagnant intestinal loop >24 h	Sum	During study intervention (until 34 weeks of gestational age)
Differential gastrointestinal diagnoses	Spontaneous intestinal perforation, Malrotation/volvulus, other	Sum	During study intervention (until 34 weeks of gestational age)
X-ray of the abdomen	Incidence rate	Mean/Median	During study intervention (until 34 weeks of gestational age)
Death	Incidence rate	Mean/Median	Third day postpartum until 40 weeks of gestational age
***Secondary outcome variables***			
Late-onset sepsis	Incidence rate	Mean/Median	During study intervention (until 34 weeks of gestational age)
Verified late-onset sepsis^b^	Incidence rate	Mean/Median	During study intervention (until 34 weeks of gestational age)
Late-onset sepsis Culture-proven	Type of pathogen	Sum	During study intervention (until 34 weeks of gestational age)
Late-onset sepsis date	Date of onset	Sum	During study intervention (until 34 weeks of gestational age)
Late-onset sepsis Peripheral line at onset	Yes/No	Mean/Median	During study intervention (until 34 weeks of gestational age)
Late-onset sepsis Central line at onset	Yes/No	Mean/Median	During study intervention (until 34 weeks of gestational age)
Use of antibiotics	Rate of prescription (number) Duration of treatment (days)	Mean/Median	During study intervention and until 40 weeks of gestational age
Length of stay	Number of days	Mean/Median	Length of stay at the neonatal intensive care unit Length of stay at homecare
Effect on microbiome	Fecal samples		1. Directly after inclusion ± 2 days 2. 14 ± 2 days gestational age 3. 34 weeks ± 2 days gestational age 4. 12 months corrected age ± 2 weeks
***Serious adverse event*** (Necrotizing enterocolitis, death and late-onset sepsis included)			
Bronchopulmonary dysplasia	Yes/No	Mean/Median	During study intervention (until 34 weeks of gestational age)
Respiratory distress syndrome	Yes/No	Mean/Median	From birth and during study intervention (until 34 weeks of gestational age)
Patent ductus arteriosus (PDA)	Yes/No	Mean/Median	From birth and during study intervention (until 34 weeks of gestational age)
Pulmonary hypertension	Yes/No	Mean/Median	During study intervention (until 34 weeks of gestational age)
Intraventricular hemorrhage	Yes/No	Mean/Median	During study intervention (until 34 weeks of gestational age)
Hydrocephalus	Yes/No	Mean/Median	During study intervention (until 34 weeks of gestational age)
Retinopathy of prematurity	Yes/No	Mean/Median	During study intervention (until 34 weeks of gestational age)
***Characteristics/covariates***			
Pregnancies	Number	Mean/Median	Baseline
Preeclampsia	Yes/No	Mean/Median	Baseline
Chorioamnionitis	Yes/No	Mean/Median	Baseline
Infection pregnancy	Number of infections	Sum	Baseline
Antibiotics during pregnancy	Period	Sum	Baseline
Mode of delivery	Vaginal or Cesarean-section	Mean/Median	Baseline
Apgar-score	At 1, 5 and 10 minutes	Mean/Median, Sum	Baseline
Gender (x/y)	Boy/girl	Mean/Median	Baseline
Multiple birth	Yes/No	Mean/Median	Baseline
Gestational age	Number (gestation week)	Mean/Median	Baseline
Birth weight	Kilograms	Mean/Median	Baseline
Birth length	Centimeters	Mean/Median	Baseline
Birth head circumference	Centimeters	Mean/Median	Baseline
Z-scores from growth chart at birth	Reference Niklasson [73] and Fenton [74]	Standard deviation	Baseline
Respiratory support Intubation	Period of treatment	Mean/Median	From birth until 40 weeks of gestational age
Respiratory support Continuous positive airway pressure (CPAP)	Period of treatment	Mean/Median	From birth until 40 weeks of gestational age
Respiratory support High-flow nasal cannula	Period of treatment	Mean/Median	From birth until 40 weeks of gestational age
Respiratory support Low-flow nasal cannula	Period of treatment	Mean/Median	From birth until 40 weeks of gestational age
Patent ductus arteriosus closure strategies Paracetamol	Period of treatment	Mean/Median	From birth until 40 weeks of gestational age
Patent ductus arteriosus closure strategies Operation	Date	Mean/Median, Sum	From birth until 40 weeks of gestational age

^a^See definition of Bell Stage >II-III under “Plans for assessment and collection of outcomes {18a}”

^b^See definition of verified late-onset sepsis under “Plans for assessment and collection of outcomes {18a}”

### Participant timeline {13}

Table 2 provides an overview of the timeline including enrolment, intervention, and follow-up.

**Table 2:**
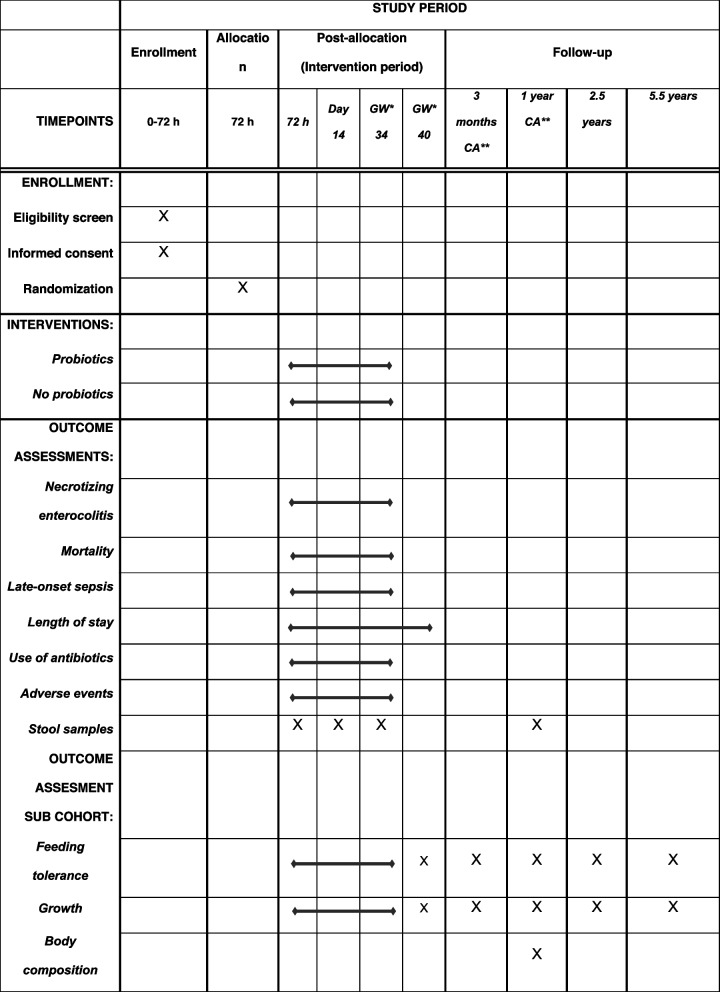
Participant timeline with checkpoints and outcome assessment during enrollment, intervention, and follow-up

*Gestational week

**Corrected age

**Fig. 1:**
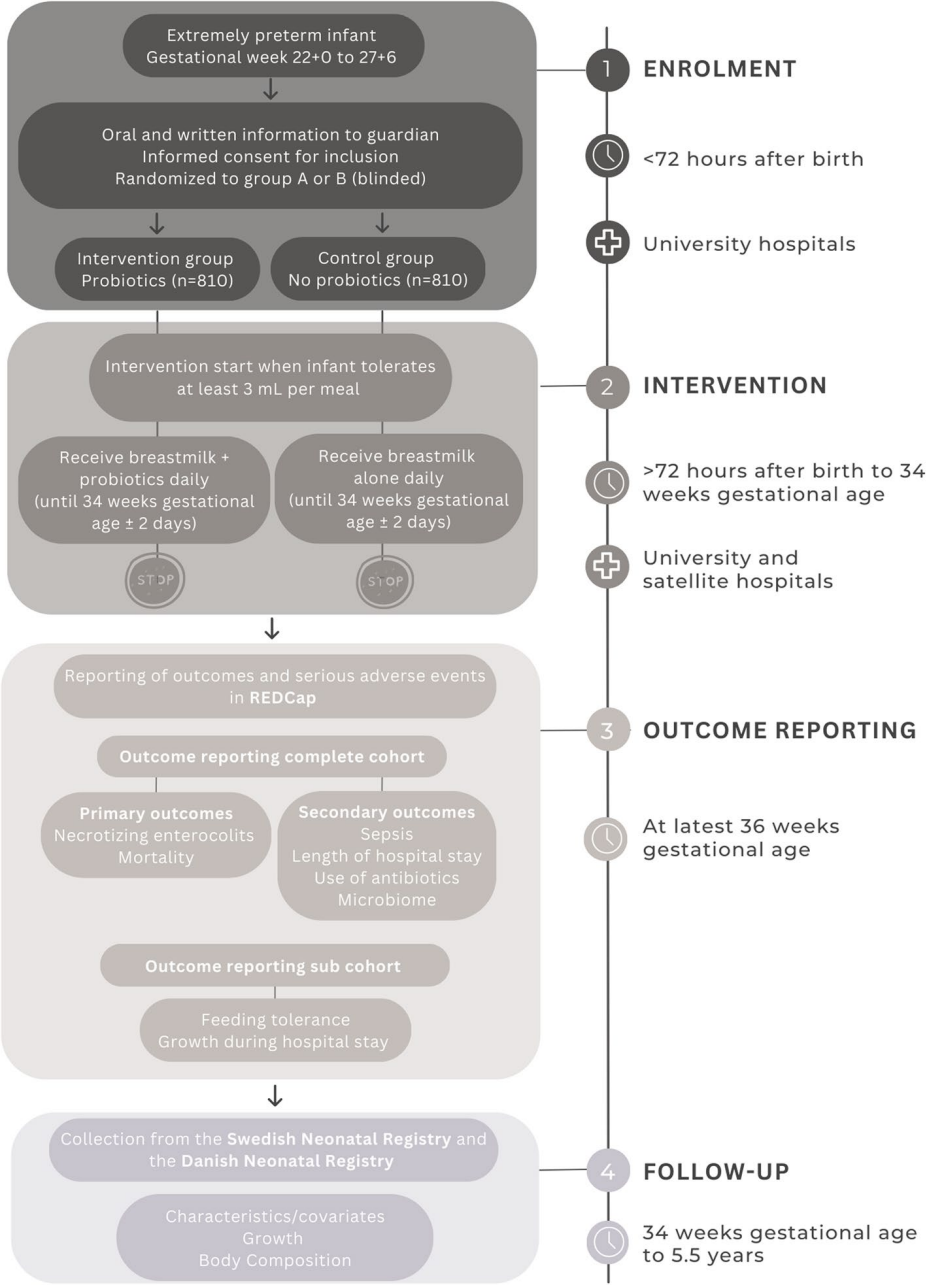
Flowchart of enrollment, intervention, outcome reporting, and follow-up

Figure 1 also provides a flowchart of enrollment, intervention, outcome reporting, and follow-up. It also depicts the participant timeline for main cohort and sub-cohort, including providing details on consent, randomization, intervention, and primary and secondary outcomes for intervention period and during follow-up.

#### Participant timeline for the sub cohort

The follow-up variables for the sub-cohort consist of anthropometric measurements at term, 3 months of age corrected for prematurity, 12 months of age corrected for prematurity, 2.5 years of age corrected for prematurity, 5.5 years of chronological age, and one body composition measurement at 3 months of age corrected for prematurity. Most infants within the sub-cohort will be followed up at the neonatal ward at Karolinska University Hospital, Sweden. If the child is not living in the Stockholm Region at follow-up, body composition will not be assessed. Growth data from the SNQ registry will still be obtained.

### Sample size {14}

The sample size calculation is based on the Swedish average incidence of NEC and mortality in extremely preterm infants, which is comparable for Denmark. During the first two to three postnatal days before the intervention is started, the mortality rate is highest among extremely preterm infants. We will calculate the mortality rate from the start of the third postnatal day, as this is the estimated time the enrolled infants will be exposed to the intervention. Based on pooled incidence rate data from SNQ data from the years of 2019, 2020, and 2021, we considered an 18% base rate of the composite outcome NEC and/or mortality, with an estimated 9% overlap. We estimated a 30% reduction, as this was considered clinically relevant and was also the NEC risk reduction rate seen in the ProPrems trial [6]. After analyzing the relative risk for the power analysis, assuming a significance level of 0.05 and 30% reduction rate with interim analysis based on a Pocock boundary at 1/3, 2/3s, and 100% of the information, the proposed enrollment of 1620 patients will have sufficient power [7] to test our hypotheses. Patient enrollment will continue until this number has been reached. Assuming an 80% consent rate, we expect to enroll 1620 infants in 3 years and 8 months.

### Recruitment {15}

To ensure adequate participant enrollment, it will be possible to enroll patients antenatally as this will ease planning of the enrollment process. Moreover, the enrollment is not only restricted to clinicians but can also be performed by trained study nurses.

To ensure that legal guardians with native languages other than Swedish understand the full extent of the trial, written consent will be available in at least seven languages (Swedish, Danish, English, Arabic, Persian, Somali and Russian). Use of a translator is recommended when needed.

## Assignment of interventions: allocation

### Sequence generation {16a}

Randomization will be done within 24 h after written consent has been obtained from the legal guardians. Allocation to either intervention or control group will be made using the randomization software, *Randomize.net*. The randomization will be stratified by center and the patient will be allocated to either group C or group D.

### Concealment mechanism {16b}

All participating hospitals will have two boxes clearly labeled as “C” and “D,” one containing probiotic sachets and one containing placebo sachets. All researchers, legal guardians, and bedside medical and nursing personnel will not know whether group C or D is receiving the probiotic supplement. The probiotic and placebo sachets are only distinguishable from one another due to being labeled with two different four-digit batch number sequences which will change with every new batch produced. Only the manufacturer of the study product and the Data Safety and Monitoring Committee (DSMC) will have insight regarding group allocation specific to these batch numbers.

### Implementation {16c}

After the legal guardians have given written consent to clinicians or study nurses, the infant will be randomly assigned to the intervention or control group. The PI at each study center will oversee group allocation via the randomization software, Randomize.net, but this may also be delegated to other research personnel. Data collection and analysis will be made using a binary treatment code to ensure continued blinding of all patients until the completion of the study.

## Assignment of interventions: blinding

### Who will be blinded {17a}

All researchers, legal guardians, and bedside medical and nursing personnel will be blinded, meaning they will not know whether group C or D receives the probiotic supplement.

### Procedure for unblinding if needed {17b}

Only the DSMC will have access to the code for blinding/unblinding. In addition, a code breaking list will be stored in a locked cabinet or equivalent and only accessible to the main PI at the central study center (Karolinska University Hospital). In case of an emergency where access to the code breaking information is required, the main PI may initiate code breaking.

## Data collection and management

### Plans for assessment and collection of outcomes {18a}

Outcome variables will mainly be collected from SNQ for the Swedish NICUs and from the Danish Newborn Quality Database (DNQD) for the Danish NICUs. Data not collected from the registries will be retrieved from patient records and collected in an electronic case report form (eCRF) documented in the electronic data capture application, REDCap. A complete list of data that will be documented in REDCap is found in Additional file 1.

The following variables will be documented in the eCRF:

- Date of randomization
- Group assignment
- Date of start of intervention
- Date of pause of intervention
- Infection during pregnancy
- Antenatal antibiotics
- If stool samples were taken (at baseline, 14 days post menstrual age (PMA), 34 weeks PMA, and 12 months corrected age)

Only verified NEC and culture-proven LOS will be used for the analysis. For NEC, this is defined as Bell Stage II-III; Bell Stage I will not be considered. Patient records will be used to exclude or add potential NEC (Bell Stage II-III) as a compliment to the SNQ and DNQ registry. The PI of each site is responsible for validating NEC and LOS diagnoses. All validation of NEC and LOS diagnoses will be made prior to unblinding at the end of study period. Validation will be documented in REDCap. All cases of NEC will be reviewed by an external expert group not involved as a researcher in the PEPS trial. This group will consist of at least two experienced neonatologists and one radiologist.

#### Diagnostic/validation of NEC

Only Bell stage II-III NEC [71] will be counted as a NEC case and considered in the analyses. Bell stage II involves clinical abdominal symptoms and radiological findings. Stage III includes the findings in stage II in addition to severe clinical condition, such as need of intubation and/or need for surgery. NEC validation must include the following:

- Abdominal symptoms in connection to NEC episode
- Radiological findings
- Possible differential diagnoses
- Abdominal surgery

#### Diagnostic/validation of LOS

LOS is defined as culture-proven sepsis via blood and/or urine culture and/or significant clinical impairment and laboratory inflammatory response, where at least one of the following must be confirmed:

1. Leucocytes (LPK) < 5 or > 20 (×10^9^ cells/L)
2. Thrombocytes (TPK) < 100 (×10^9^ cells/L)
3. C-reactive protein (CRP) > 15 mg/L

#### Data collection and assessment for sub-cohort

Data on postnatal growth (Table 3) during the intervention period will be registered as per clinical routine in the nutritional calculation software, Nutrium (nutrium.se). All growth data from the intervention period will be retrieved from Nutrium. Growth data during long-term follow-up will be collected from SNQ and/or medical records (sub-cohort).

**Table 3:** Description of outcomes with analysis metric, aggregation method, and time points in sub-cohort

Outcome variables sub cohort			
*Outcome variables - feeding tolerance*	*Analysis metrics*	*Method of aggregation*	*Time point*
Enteral nutrition	Number of days until full enteral feeds	Mean/Median	During study intervention (until 34 weeks of gestational age)
Parental nutrition	Number of days with parental nutrition	Mean/Median	During study intervention (until 34 weeks of gestational age)
Interruptions of enteral nutrition	Number of enteral nutrition interruptions >8 h	Mean/Median	During study intervention (until 34 weeks of gestational age)
Enteral nutrition decreased due to clinical instability	Number of EN decrease >50%	Mean/Median	During study intervention (until 34 weeks of gestational age)
Use of breastmilk fortifiers	Number of days until breastmilk fortifiers is induced	Mean/Median	During study intervention (until 34 weeks of gestational age)
Feeding tube requirement	Number of days requiring a feeding tube	Mean/Median	During study intervention and until 40 weeks of gestational age
Macronutrient intake	Amount of macronutrients given (kcal/kg/day)	Mean/Median	During study intervention (until 34 weeks of gestational age)
Micronutrient intake	Amount of micronutrients given (μg or mg/kg/day)	Mean/Median	During study intervention (until 34 weeks of gestational age)
***Outcome variables - postnatal growth***			
Attaining birth weight	Number of days	Mean/Median	After attaining birth weight after normal initial weight loss postpartum
Body weight	Kilograms	Mean/Median	1. Once every week during study intervention 2. 40 weeks of gestational age 3. 3 months of corrected age 4. 12 months of corrected age 5. 2.5 years of corrected age 6. 5.5 years of age
Body length	Centimeters	Mean/Median	1. Once every week during study intervention 2. 40 weeks of gestational age 3. 3 months of corrected age 4. 12 months of corrected age 5. 2.5 years of corrected age 6. 5.5 years of age
Head circumference	Centimeters	Mean/Median	1. Once every week during study intervention 2. 40 weeks of gestational age 3. 3 months of corrected age 4. 12 months of corrected age 5. 2.5 years of corrected age 6. 5.5 years of age
Growth chart *Z*-score	Reference Niklasson [73] and Fenton [74]	Standard deviation	1. Once every week during study intervention 2. 40 weeks of gestational age 3. 3 months of corrected age 4. 12 months of corrected age 5. 2.5 years of corrected age 6. 5.5 years of age
Body composition	Fat mass, fat-free mass (%)	Average	3 months corrected age
***Characteristics/covariates***			
Use of medical steroids	Period of treatment	Mean/Median	From birth until 5.5 years of age
Use of tetracyclines	Period of treatment	Mean/Median	From birth until 5.5 years of age
Type of enteral nutrition mothers’ own milk, donated breastmilk, formula	Percentages (%)	Proportion	During study intervention (until 34 weeks of gestational age)
Breastmilk fortification	Type	Proportion	During study intervention (until 34 weeks of gestational age)
Breastfeeding at discharge	Full, partly, none	Proportion	Discharge from neonatal intensive care unit/homecare

Daily nutritional assessment of enteral and parenteral nutrition during the intervention period will be documented in Nutrium. These assessments include all macro- and micronutrient intake including type, amount, and strategy of enteral and parenteral feeds. Data on breastmilk fortification and interruptions of enteral feeds will also be documented and collected from Nutrium.

Assessment of body composition will be made at 3 months corrected age using air displacement plethysmography (PeaPod). This is considered “the gold standard” for measuring body composition, including fat, fat-free mass, and bone density, for infants weighing below 8 kg [72]. It applies whole-body plethysmography which is a fast, reliable, and non-invasive assessment. Assessment of body composition will be documented and retrieved from patient records.

### Plans to promote participant retention and complete follow-up {18b}

Hospital discharge before 34 weeks of gestational age is very uncommon among extremely preterm infants. Therefore, the intervention will be completed during hospital admission and the drop-out rate is expected to be none or minimal. Legal guardians will be given robust information about the study both before inclusion and following enrollment, which is expected to promote study retention.

Patients will be included at a university hospital that cares for extremely preterm infants from birth, but may be relocated to satellite hospitals during the intervention. In the event of relocation, the initial university hospital and the main study coordinator are responsible for giving the cooperating satellite hospital adequate information about the study. The receiving satellite hospital will receive standardized written information, including general information about the study, how to prepare and administer the study product, and information about stool sampling. The PI for each university hospital is responsible for completing the CRF and other administrative data collection in REDCap.

Stool sampling at 12 months corrected age will be taken by the legal guardians at home if infants are not followed up at a university hospital. Instructions for legal guardians will be sent out by the neonatal follow-up clinic when informing parents about routine hospital appointments. Neonatal follow-up clinics will be given sufficient information about stool sampling before the first included infant reaches 12 months corrected age.

### Data management {19}

Data will be collected and maintained with the electronic data capture application, REDCap. REDCap is a secure web platform for building and managing online databases and surveys. Data will be accessible for all research group members but not to unauthorized personnel. Members of the research team at every site will only have access to data from their own hospital. The PI and other members from the main research group at Karolinska will have access to data collected at all hospitals. All researchers will be blinded during the study and analyses.

REDCap will be accessible to all university hospitals taking part in the research project. The software enables very robust traceability of the research output, including an audit trail that traces data entry and editing. Access to what data can be viewed and modified can be customized for each person within the research project. The data is stored automatically at two different servers around Stockholm which are backed up every hour. Data will be analyzed using the statistical analysis software, STATA.

### Confidentiality {27}

Patients will be pseudonymized and obtain a Study-ID upon inclusion. A patient identification log will be available in REDCap. Only core researchers with will have access to the identification log, and their access will be limited to that of their own university hospital. The PI and other members from the main research group at Karolinska will have access to all patient identification logs. The patient identification log will only have information about if the patient has been allocated to group C or D, so this information will still be blinded.

Management of data will be done according to the European Union’s General Data Protection Regulation (GDPR) guidelines. No unauthorized person will have access to personal data and those with access will be bound by professional secrecy. Region Stockholm is responsible for the participants’ personal data.

Legal guardians have the right to access the personal information handled in the study and correct errors if necessary. They can also request the deletion and restriction of personal data; however, this right does not apply when the data is necessary for the research in question. Legal guardians who are not content with how personal data is processed can submit a complaint to Swedish Authority for Privacy Protection.

The analysis and publishing of the results will be performed at a group level, meaning that no individuals can be traced to their data. To ensure the safety and integrity of the patients and quality of collected data, the study will be conducted in accordance with the study protocol, ICH-GCP E6 (R2) and the Declaration of Helsinki. The trial is designed in accordance with recommendations for interventional trials [68] and Consolidated Standards for Reporting of Trials (CONSORT) guidelines.

### Plans for collection, laboratory evaluation, and storage of biological specimens for genetic or molecular analysis in this trial/future use {33}

For the complete cohort, stool samples to identify possible differences in microbiome composition will be collected at the following timepoints:

- Directly after inclusion or at the timepoint when infant first produces stool
- 14 ± 2 days gestational age
- 34 weeks ± 2 days gestational age
- 12 months corrected age ± 2 weeks

The stool samples will be collected by a nurse and stored immediately in −20°C and/or −80°C. They should be transferred frozen to a −80°C freezer at the hospital if available or at the regional biobank as soon as possible. All collected stool samples will be moved and registered at KI Biobank at Karolinska Institutet in Solna, Sweden, and handled in accordance with current biobank regulations at the end of the trial. A multicenter agreement between all biobanks included in the PEPS trial has been established. All samples will be coded with the participants’ study-ID and stored securely and separately to prevent unauthorized access and hold pseudonymization until the results have been finalized. Stool management, microbiological sampling, and complete sequencing will be done in collaboration with the Centre for Translational Microbiome Research at KI.

## Statistical methods

### Statistical methods for primary and secondary outcomes {20a}

Data will be analyzed as both per protocol and intention to treat (ITT). The primary analysis will be analyzed and reported as ITT.

#### Primary aim 1 and secondary aims 1–3

In the initial analysis, we will compare baseline characteristics of the study population between allocation groups to ensure that balance was achieved by the randomization. We will report the mean (standard deviation [SD]) or median (first quartile, third quartile) for continuous variables, and count and percentages for categorical variables. The primary analyses will be based on the ITT population including all subjects that were randomized in the study. A crude and a multivariable-adjusted standardized logistic regression will be used to calculate the relative risk (RR) and 95% CI to estimate the effect of probiotic supplementation on the risk of NEC and/or mortality (primary aim) as well as NEC, total mortality, and LOS separately (secondary aims 1–3). The randomization will be stratified per site. Thus, the analysis will adjust for this variable before standardization. A per-protocol analysis accounting for non-compliance will also be performed. All data analyses will be performed using the STATA and R software. A two-sided *p*-value of 0.05 will be considered significant evidence of the effect of the intervention.

#### Secondary aims 4 and 5

The effect of probiotic supplementation on the rate of prescription and duration of antibiotic use during hospital stay (secondary aim 4) and duration of hospital stay (secondary aim 5) will be analyzed using standardized logistic regression and linear regression model.

#### Secondary aim 6

The effect of probiotic supplementation on gut microbiome will be investigated by comparing the alpha and beta diversity of the microbiota samples. Alpha diversity measures the mean diversity within a sample while beta diversity involves a comparison of diversities between samples and will be displayed as a principal coordinate or component analysis plot.

#### Tertiary aims 1–2

The effect on feeding tolerance (tertiary aim 1) and postnatal growth (tertiary aim 2) will be analyzed using standardized logistic regression and linear regression models.

### Interim analyses {21b}

The DSMC will carry out interim analysis based on a Pocock boundary for safety and efficacy analyses at the time points when 1/3 (*n*=540), 2/3 (*n*=1080), and 3/3 (*n*=1620) of patients have been included. All interim analyses will use the same method as described for primary aims and secondary aims 1-3. A two-sided *p*-value of 0.02205 will be considered significant evidence of the effect of the intervention for the first interim analysis, 0.3794 for the second, and 0.05 at full information accrual.

### Methods for additional analyses (e.g., subgroup analyses) {20b}

All primary, secondary, and tertiary outcomes alongside baseline characteristics and potential covariates are described above in section Outcomes {12} and Table 1 and Table 3. We will perform a sensitivity analysis for variables, including but not limited to sex, site, gestational age, birth weight, and mode of delivery.

Subgroup analyses for site, sex, and gestational age will be performed, the latter consisting of both infants born between 22 weeks + 0 days to 24 weeks + 6 days and those born between 25 weeks + 0 days to 27 weeks + 6 days.

For the sub-cohort, we will perform subgroup analyses based on type of enteral nutrition (volumes of breastmilk intake) and amount of macro- and micronutrients in relation to growth, body composition, and enteral feeding tolerance. The cut-off value for macro- and micronutrient intake will be based on ESPGHAN’s recommendation for parenteral and enteral intake (below recommended intake, in line with recommendation, or above recommended intake) [48].

Additional subgroup analyses may be performed if empirical data show unexpected patterns, such as potentially systematical differences in baseline characteristics, outcome, or adverse events (AE) between groups. All planned subgroup analyses will clearly be stated in the study report.

### Methods in analysis to handle protocol non-adherence and any statistical methods to handle missing data {20c}

A per-protocol analysis accounting for non-compliance will be performed. Despite this, there is no anticipation of a significant degree of non-adherence and/or missing data, as the intervention will only be carried out during hospitalization and most outcome data after discharge will be generated from the SNQ and DNQD registry. If data is missing from a registry extract, patient records will be searched to complete data collection.

### Plans to give access to the full protocol, participant-level data, and statistical code {31c}

The full study protocol is available upon request. However, participant-level data are not available without ethical approval, in order to protect the patient integrity.

## Oversight and monitoring

### Composition of the coordinating center and trial steering committee {5d}

The main PI for the coordinating center is responsible for overall staff management, including funding and time and resource management.

The main research team consists of the main PI, three senior researchers and one doctoral student responsible for planning, implementation, execution, and analysis of the research. Apart from one, all main researchers are physically located at the coordinating center.

At the coordinating center, there will be a study coordinator with 100% dedication to the project who will help with inclusion and data collection, sampling, biobanking, and overall trial contact managing.

At each university hospital site, there will be at least one study nurse working part-time to manage the inclusion of patients, data collection, and stool sampling. Each site will have a responsible PI who can delegate responsibilities to other clinicians, such as obtaining informed consent. These hospital-site-specific groups will have close day-to-day contact to manage planning of inclusion and other study events, such as stool sampling.

At each satellite hospital site, there will be one main contact and at least one nurse to manage the continuation of the intervention.

The trial steering committee will consist of ten senior neonatologists who are also PIs at each respective hospital. Meetings coordinated by the coordinating center will be arranged at least once per semester during the study and when analyzing and publishing results.

### Composition of the data monitoring committee, its role and reporting structure {21a}

A DSMC was established consisting of two experienced clinicians with expertise in neonatology and research methodology and one biostatistician. All cases of SAEs, SUSARs, and other AEs that clinicians deem concerning and report will be reviewed by the committee. Criteria for stopping the ongoing intervention will be decided by the committee. Information about how SUSARs are reported is described in paragraph below.

### Adverse event reporting and harms {22}

#### Adverse events

AEs are typically defined as unwanted medical events, such as side effects or altered laboratory findings, in a patient who has received the study product—no matter whether it is associated with the intervention given or not. Extremely preterm infants are expected to suffer from various morbidities and may require respiratory support, such as intubation and continuous positive airway pressure (CPAP), for many weeks during the intervention. This will likely influence laboratory findings, such as variations in blood gas tests taken in accordance with standard care. Therefore, medical findings in such tests will not be collected in the eCRF. The same principle applies to other tests as part of standard care, such as an ultrasound, with findings that do not conform to an SAE. If the clinicians are unsure whether to document an AE, this must be discussed with the site PI. Uncertainties about the documentation of AEs can be discussed with main PI and during trial steering committee meetings.

#### Serious adverse events

SAEs are typically defined as unwanted medical events that can result in any of the following outcomes: death, life-threatening medical condition, extended hospitalization, and permanent or significant disability. Extremely preterm infants are vulnerable and at high risk for complications, particularly during the first postnatal weeks. Thus, mortality and various morbidities are expected in the study population. In this trial, we will define expected SAEs as events that occur in over 5% of the study population in accordance with current clinical data from SNQ.

The following SAE are expected in the study population:

- Infections; LOS, meningitis, cytomegalovirus (CMV) infection
- Gastrointestinal; NEC, spontaneous intestinal perforations, volvulus, ileus, other malrotation
- Lung function; bronchopulmonary dysplasia (BPD), respiratory distress syndrome
- Circulation; patent ductus arteriosus (PDA), pulmonary hypertension
- Brain function; intraventricular hemorrhage (IVH), periventricular leukomalacia (PVL), hydrocephalus
- Retinopathy of prematurity (ROP)
- Death

#### Reporting serious adverse events

All SAEs will be recorded in the eCRF and do not need to be documented immediately. However, to be able to carry out interim analyses, all SAEs must be documented no later than 2 weeks after the intervention has ended for each patient.

#### Suspected unexpected serious adverse events

SUSARs are typically defined as a reaction or event that is unexpected, serious, and suspected to be caused by the intervention. In this trial, death is defined as an SAE. However, when a SUSAR occurs in connection to an event that later causes death, this must be reported.

#### Reporting SUSARs

Investigators are required to report SUSARs to the main PI as soon as possible and no later than 24 h following the incident. The initial report must be made by phone.

A complementary written report must be completed immediately using the data collection instrument in REDCap entitled, “reporting of SUSAR.”

In the event of code breaking in connection to a SUSAR, blinding must still be preserved during the ongoing trial. The oral or written report should therefore not contain information about regarding whether the patient was receiving the probiotic supplement or not.

#### Management and follow-up of SUSAR

The main PI at Karolinska University Hospital will oversee the reporting of SUSARs to the DSMC immediately after receiving the report. The DSMC will also have accesses to the written report in REDCap. The DSMC will investigate and follow-up all SUSAR events.

### Frequency and plans for auditing trial conduct {23}

As most data collection is registry-based, the need for auditing trial conduct regarding quality of data collection is limited. As such, auditing of trial conduct will not be undergone. The main research group will have access to all data collection made at all hospitals in REDCap. Site monitoring will thus mainly be made by auditing data collection, such as missing variables, in REDCap. Potential need for revision of trial conduct will mainly be handled by the trial steering committee meeting.

### Plans for communicating important protocol amendments to relevant parties (e.g., trial participants, ethical committees) {25}

The main PI and the study coordinator will be responsible for communicating any relevant amendments to the study protocol to all relevant parties. All protocols will be dated and version managed. Potential amendments to the study protocol will also be documented in a separate report file to ease the identification of alterations. If any major changes are made to the study protocol, an amendment will be sent to the Swedish Ethical Review Authority and the Danish Ethical Review Authority if applicable (complete cohort).

### Dissemination plans {31a}

The main PI is responsible for finalizing the study report. The results will be published in scientific peer-reviewed journals and presented at national and international congresses and scientific meetings.

## Discussion

To our knowledge, the PEPS trial is the first sufficiently powered, placebo-controlled RCT evaluating the effect of probiotic supplementation in preventing NEC, total mortality, and other secondary and tertiary endpoints in extremely preterm infants. The results will provide high-quality evidence and likely inform supplementation recommendations in this population. Furthermore, this trial may also add new knowledge on the effect of probiotic supplementation on feeding tolerance and growth, microbiome diversity and development, and body composition in relation to nutritional intake. We believe these results will provide important in-depth knowledge in the context of the results from the main trial.

Despite extensive undertaking of probiotic trials in preterm infants, many challenges have been encountered. Firstly, these trials have often been small in scale and underpowered to effectively evaluate efficacy among extremely preterm infants. Additionally, there has been inconsistency in the selection of outcomes and study populations, rendering studies incomparable. Any combined analyses of these trials have further been limited by their heterogeneity due to considerable variation in investigated probiotic strains and dosages, rendering it difficult to draw clear conclusions [17]. This knowledge gap is especially notable in the case of extremely preterm infants, who are at the highest risk for conditions, such as NEC, sepsis, and mortality. While studies have frequently involved this group of patients, they often fail to specifically examine the effects of probiotics in this population. As a result, there is frequently an inadequate number of patients included and inconclusive groups from the resulting subgroup analysis.

A recent systematic review and meta-analysis of 60 trials with data on 11,156 infants found a 46% lower risk of NEC and 23% lower risk of mortality among very preterm or low birth weight infants following probiotic supplementation [43]. However, these comparisons only provided low or moderate certainty of evidence and the corresponding estimates for extremely preterm or extremely low birth weight infants were not statistically significant for NEC and total mortality. The review further underlined the need for large, high-quality trials, particularly for this population of extremely preterm infants.

Although the prominent ProPrems trial conducted on infants born <32 weeks of gestational age reported a 54% reduced risk of NEC of Bell stage II or more following provision of the combination preparation of *B. infantis Bb-02*, *B. lactis Bb-12*, and *Str. thermophilus TH4*, this trial was underpowered for extremely preterm infants [12]. Nevertheless, the 2020 ESPGHAN position paper concluded that either *L. rhamnosus GG ATCC53103* or the combination of *B. infantis Bb-02*, *B. lactis Bb-12*, and *Str. thermophilus TH4* used in the ProPrems trial can be given to infants with birth weight <1500 g, which strengthens the investigation of this product in our study population [15]. Despite national uncertainty regarding the efficacy of probiotics in extremely preterm infants, the high incidence of NEC and mortality warrants the need for future research. Through providing sufficiently powered, high-quality evidence on the potential effectiveness of probiotic supplementation in extremely preterm infants, the results of this study will have implications on both national and international guideline development and clinical practice, thereby strengthening the evidence-based care of this vulnerable patient population going forward.

### Supplementary Information

**Additional file 1.** Data Collection form in REDCap

## Data Availability

The datasets used and/or analyzed during the current study are available from the corresponding author on reasonable request.

## Abbreviations

AE: Adverse event
BPD: Bronchopulmonary dysplasia
CPAP: Continuous positive airway pressure
CRF: Case report form
CRP: C-reactive protein
DNQD: Danish Newborn Quality Database
DSMC: Data Safety and Monitoring Committee
ESPGHAN: European Society for Paediatric Gastroenterology, Hepatology and Nutrition
HC: Head circumference
IVH: Intraventricular hemorrhage
LOS: Late-onset sepsis
NEC: Necrotizing enterocolitis
NICU: Neonatal intensive care unit
PVL: Periventricular leukomalacia
PI: Principal investigator
RCT: Randomized controlled trial
RDS: Respiratory distress syndrome
ROP: Retinopathy of prematurity
SAE: Serious adverse event
SNQ: Swedish Neonatal Quality Register
SUSAR: Suspected unexpected serious adverse reaction
