# Supplementary material for: Probiotic supplementation and risk of necrotizing enterocolitis and mortality among extremely preterm infants—the Probiotics in Extreme Prematurity in Scandinavia (PEPS) trial: study protocol for a multicenter, double-blinded, placebo-controlled, and registry-based randomized controlled trial
Source: Trials. 2024 Apr 12;25:259. doi: 10.1186/s13063-024-08088-8 (PMC11015611; doi:10.1186/s13063-024-08088-8)
Supplement: Supplementary file 1 — Additional file 1. Data Collection form in REDCap [file 13063_2024_8088_MOESM1_ESM.pdf]

Case Report Form (CRF)

Record ID

____

Study ID

____

Date of randomization

____
(YY-MM-DD)

Group assignment

◯ A
◯ B
(A or B)

Start date of intervention

____

Intervention interrupted
Note if intervention has been interrupted during study intervention period.

Add description for interrupting intervention.

If numerous interruptions: add 1, 2, 3 and so on before every interruption period

____

Infection during pregnancy

◯ Yes
◯ No

Antenatal antibiotics

◯ Yes
◯ No
(Note Yes if mother has been given antibiotics during pregnancy)

Stool sample at baseline

◯ Yes
◯ No
(As soon as possible after inclusion/randomization)

Stool sample at 14 (+/- 2 days)

◯ Yes
◯ No

Stool sample at 34 weeks gestational age (+/- 2 days)

◯ Yes
◯ No

Stool sample at 12 months corrected age

◯ Yes
◯ No

Comments
Add potential comments to data entery in CRF

____

Case Report Form (CRF) - sub cohort

Study ID	__________________________________
Bolus feeds	◯ Yes<br>◯ No
Period of treatment with bolus feeds<br>Document as YY-MM-DD - YY-MM-DD.<br>Or document if the patients has always been given bolus feeds.	__________________________________
Continious feeds	◯ Yes<br>◯ No
Period of treatment with continious feeds<br>Document as YY-MM-DD - YY-MM-DD.<br>Or document if the patients has always been given continious feeds.	__________________________________
Interruptions of enteral feeds for >8 hours	__________________________________

Verification of NEC

Study ID	______________________
Episode 1<br>Abdominal sympthoms<br><br>INCLUDE DATE OF EVENT	______________________<br>(Description)
Episode 1<br>Radiological findings<br><br>INCLUDE DATE OF EVENT	______________________<br>(Description)
Episode 1<br>Differential diagnoses	______________________<br>(SIP, malrotation, volvulus, other?)
Episode 1<br>Operation with NEC findings	◯ Yes<br>◯ No
Episode 1<br>Verified NEC	◯ Yes<br>◯ No
Episode 2<br>Abdominal sympthoms<br><br>INCLUDE DATE OF EVENT	______________________
Episode 2<br>Radiological findings<br><br>INCLUDE DATE OF EVENT	______________________
Episode 2<br>Differential diagnoses	______________________
Episode 2<br>Operation with NEC findings	◯ Yes<br>◯ No
Episode 2<br>Verified NEC	◯ Yes<br>◯ No

Episode 3
Abdominal sympthoms

____

INCLUDE DATE OF EVENT

Episode 3
Radiological findings

____

INCLUDE DATE OF EVENT

Episode 3
Differential diagnoses

____

Episode 3
Operation with NEC findings

○ Yes
○ No

Episode 3
Verified NEC

○ Yes
○ No

Episode 4
Abdominal sympthoms

____

INCLUDE DATE OF EVENT

Episode 4
Radiological findings

____

INCLUDE DATE OF EVENT

Episode 4
Differential diagnoses

____

Episode 4
Operation with NEC findings

○ Yes
○ No

Episode 4
Verified NEC

○ Yes
○ No

Episode 5
Abdominal sympthoms

____

INCLUDE DATE OF EVENT

Episode 5
Radiological findings

INCLUDE DATE OF EVENT

____

Episode 5
Differential diagnoses

____

Episode 5
Operation with NEC findings

◯ Yes
◯ No

Episode 5
Verified NEC

◯ Yes
◯ No

Comments

Use for comments or for documenting more potential NEC episodes

INCLUDE DATE OF EVENT

____

Verification of Sepsis

Study ID	__________________________________
Sepsis episode 1<br>LPK < 5 or > 20 (x109 cells/L)	◯ Yes<br>◯ No
Sepsis episode 1<br>TPK < 100 (x109 cells/L)	◯ Yes<br>◯ No
Sepsis episode 1<br>CRP > 15 mg/L	◯ Yes<br>◯ No
Verified sepsis<br>Incidence of sepsis is defined as culture proven sepsis including positive blood, urin - and/or urin, clinical impairment and laboratory inflammatory response were at least one of the three above is confirmed	◯ Yes<br>◯ No
Sepsis episode 2<br>LPK < 5 or > 20 (x109 cells/L)	◯ Yes<br>◯ No
Sepsis episode 2<br>TPK < 100 (x109 cells/L)	◯ Yes<br>◯ No
Sepsis episode 2<br><br>CRP > 15 mg/L	◯ Yes<br>◯ No
Sepsis episode 2<br><br><br>Verified sepsis<br>Incidence of sepsis is defined as culture proven sepsis including positive blood, urin - and/or urin, clinical impairment and laboratory inflammatory response were at least one of the three above is confirmed	◯ Yes<br>◯ No
Sepsis episode 3<br>LPK < 5 or > 20 (x109 cells/L)	◯ Yes<br>◯ No
Sepsis episode 3<br>TPK < 100 (x109 cells/L)	◯ Yes<br>◯ No
Sepsis episode 3<br>CRP > 15 mg/L	◯ Yes<br>◯ No

Sepsis episode 3<br><br>Verified sepsis<br>Incidence of sepsis is defined as culture proven sepsis including positive blood, urin - and/or urin, clinical impairment and laboratory inflammatory response were at least one of the three above is confirmed	◯ Yes<br>◯ No
Sepsis episode 4<br>LPK < 5 or > 20 (x109 cells/L)	◯ Yes<br>◯ No
Sepsis episode 4<br>TPK < 100 (x109 cells/L)	◯ Yes<br>◯ No
Sepsis episode 4<br>CRP > 15 mg/L	◯ Yes<br>◯ No
Sepsis episode 4<br><br><br>Verified sepsis<br><br>Incidence of sepsis is defined as culture proven sepsis including positive blood, urin - and/or urin, clinical impairment and laboratory inflammatory response were at least one of the three above is confirmed	◯ Yes<br>◯ No

Reporting Serious Adverese Events (SAE)

Study ID	__________________________
Death	◯ Yes<br>◯ No
	__________________________
Verified NEC	◯ Yes<br>◯ No
	__________________________
Verified sepsis	◯ Yes<br>◯ No
	__________________________
RDS	◯ Yes<br>◯ No
	__________________________
BPD	◯ Yes<br>◯ No
	__________________________
PDA	◯ Yes<br>◯ No
	__________________________
PPHN	◯ Yes<br>◯ No
	__________________________
IVH	◯ Yes<br>◯ No
	__________________________

Hydrocephalus	◯ Yes<br>◯ No
	______________________
ROP	◯ Yes<br>◯ No
	______________________
Gastrointestinal diagnoses (volvulus, illeus, SIP, malrotation, other)	______________________<br>(description)
	______________________
Comments	______________________

Reporting Suspected unexpected serious adverse event (SUSAR)

Study ID

____

Patients name

____
(First and second name)

Patients Social Security Number

____
(Personnummer/reservnummer)

Date of event (SUSAR)

____

Time of event (if appliable)

____

Place of event

____

Description of event
DO NOT ADD INFORMATION ABOUT GROUP ALLOCATION EVEN IF CODE BREAKING HAS ACCOURED

____

Screening Log

Date of screening	__________________________________
Gestational age	__________________________________<br>(Week + day (For example 26+3))
Meets inclusion criteria	◯ Yes<br>◯ No
If previous answers was "No". Please add description why inclusion criterias were not meet.	__________________________________
Included in PEPS-trial	◯ Yes<br>◯ No
If previous answers was "No" even though inclusion criteria were met. Add description why the patient was not included.<br>For example: parents did not give consent	__________________________________
STUDY ID<br>If included (N-NNN)	__________________________________
Other information	__________________________________<br>(if appliable)

Patient Identification Log

STUDY ID

____
(Add study id (N-NNN))

Full Name

____
(First name and last name)

Social Security Number

____
("personnummer eller reservnummer")

Date of randomization

____
(YY-MM-DD)

Group allocation

- ◯ A
- ◯ B

Including hospital

- ◯ Karolinska UH Solna
- ◯ Karolinska UH Huddinge
- ◯ Linköping UH
- ◯ Lund UH
- ◯ Sahlgrenska UH
- ◯ Umeå UH
- ◯ Uppsala UH
- ◯ Aarhus UH
- ◯ Copenhagen UH
- ◯ Glostrup UH
- ◯ Odense UH
- ◯ Aalborg
